# Correlation between albuminuria and interstitial injury marker reductions associated with SGLT2 inhibitor treatment in diabetic patients with renal dysfunction

**DOI:** 10.1186/s40001-022-00737-5

**Published:** 2022-08-06

**Authors:** Saeko Sato, Kaori Takayanagi, Taisuke Shimizu, Koichi Kanozawa, Takatsugu Iwashita, Hajime Hasegawa

**Affiliations:** 1 Department of Nephrology and Hypertension, Saitama Medical Center, Saitama Medical University, 1981 Kamoda, Kawagoeshi, Saitama, 350-8550 Japan; 2 Ishikawa Kinenkai Kawagoe Ekimae Clinic, 16-23, Wakitahoncho, Kawagoeshi, Saitama, 350-1123 Japan

**Keywords:** Diabetic tubulopathy, Fibrosis, MCP-1, Proteinuria, Type 2 diabetes, N-Acetyl-β-D-glucosaminidase

## Abstract

**Background:**

We investigated the effects of sodium–glucose cotransporter 2 inhibitor (SGLT2i) administration focusing on its involvement in tubulo-interstitial disorders in diabetic kidney.

**Methods:**

Enrolled patients with diabetic kidney disease received a mean dose of 52.3 mg of an SGLT2i (ipragliflozin) daily. Blood and urine were sampled at 0, 1, and 12 months (M).

**Results:**

Non-renal-dysfunction patients (NRD: baseline eGFR ≥ 60 mL/min/1.73 m^2^, *n* = 12) and renal-dysfunction patients (RD: baseline eGFR < 60 mL/min/1.73 m^2^, *n* = 9) were analyzed separately. The median urine albumin-to-Cr ratio (ACR) was significantly decreased at 1 M in both groups (NRD: 163.1 at 0 M vs 118.5 mg/g Cr at 1 M, RD: 325.2 at 0 M vs 136.0 mg/g Cr at 1 M). In the RD, but not the NRD group, reduction of urine monocyte chemotactic protein-1 (MCP-1) by SGLT2i showed a significant difference between high-responders (HR: − 25.7 ± 11.4%) and low-responders (LR: 59.2 ± 17.0%), defined by ACR reduction at 1 M. Univariate analysis showed a significant correlation between the reduction of ACR and MCP-1 (*R* = 0.683, *p* = 0.042) in RD.

**Conclusion:**

SGLT2i exerted an anti-albuminuric effect regardless of the presence/absence of renal dysfunction. However, the anti-albuminuric effect of SGLT2i in patients with renal dysfunction appears more closely associated with amelioration of tubulo-interstitial disorders compared to patients without renal dysfunction.

## Introduction

Diabetic kidney disease (DKD) including diabetic nephropathy (DN) remains the leading cause of end-stage renal disease, and its management is an important issue in clinical nephrology. In diabetic nephropathy, glomerular hypertension with glomerular hyperfiltration (GHF) is mentioned as one of the principal factors which induces glomerular disturbance with various metabolic abnormalities, inflammation and body fluid retention. Abnormal tubulo-glomerular feedback (TG feedback) in particular is recognized as a characteristic hemodynamic feature in diabetic nephropathy [1]. Increased sodium reabsorption in the proximal tubules results in the sodium delivery to the macula densa being disproportionally decreased in relation to the GFR, leading to pathologically dilated afferent arterioles and finally causing sustained GHF, which are known as tubular hyperfiltration [2]. Tubular hyperfiltration is thought to play a principal role in the development of GHF and resultant glomerular disorders in DN [3].

Independent of glomerular damages, tubulo-interstitial disorders including tubular atrophy, interstitial fibrosis, and a reduced number of peritubular capillaries are also observed in diabetic kidneys; the presence of such lesions is known as diabetic tubulopathy [4–6]. A decreased partial pressure of oxygen has been reported in the cortical areas of diabetic kidneys, due to the increased oxygen demand, as required for glucose metabolization, and decreased oxygen supply due to a decreased number of peritubular capillaries [6, 7]. The resultant ischemia stimulates tubular cells and fibroblasts, leading to a transforming growth factor (TGF)-ß-dependent or -independent extracellular matrix expansion, resulting in fibrosis [6, 8]. These various pathological settings are together involved in the progression of tubulopathy.

Sodium–glucose cotransporter 2 (SGLT2) inhibitor, a widely used oral anti-hyperglycemic agent, inhibits tubular sodium and glucose reabsorptions [9]. Several renoprotective effects of SGLT2 inhibitors are known [10–12], including the correction of GHF via a reduction of sodium delivery to the macula densa [13]. Additionally, recent work has revealed that SGLT2 inhibitors improve tissue hypoxia by reducing the energy consumption due to reduced glucose accumulation, resulting in an improvement of tubulopathy [14]. Less accumulation of advanced glycation end products (AGEs) is also associated with the improvement of tubulopathy [15, 16]. Indeed, SGLT2 inhibitor reduces urinary excretions of kidney injury molecule 1 (Kim-1), liver-type fatty acid binding protein (L-FABP), N-acetyl-β-D-glucosaminidase (NAG), and other biomarkers relevant to tubulo-interstitial disorders [6]. It has been also suggested that tubulo-interstitial disorders have a key role in the development of albuminuria in type-1 DM [17].

In general, for progressive exacerbation of renal diseases, tubulo-interstitial disorders have the same or greater pathological significance as glomerular disorders. We attempted to verify the possibility of ameliorating tubulo-interstitial disorders by administering SGLT2 inhibitor by analyzing the changes in albuminuria reduction and tubulo-interstitial markers associated with the administration of this drug. Here, we observed that (1) an SGLT2 inhibitor exerted anti-albuminuric effects regardless of the presence/absence of renal dysfunction, and (2) the reduction of albuminuria was correlated with the reduction of monocyte chemotactic protein-1 (MCP-1) excretions in patients with renal dysfunction. We thus speculate that the underlying mechanisms of albuminuria reduction of SGLT2 inhibitors may differ according to the presence or absence of renal dysfunction, and that the amelioration of tubulo-interstitial disorders might be an important aspect of the anti-albuminuric effect of SGLT2 inhibitors in patients with renal dysfunction.

## Patients and methods

### Study design

The population for the analysis in this study was originally planned as a prospective controlled open-label trial (#1062 study of Saitama Medical Center, Saitama Medical University) of an SGLT2 inhibitor and sulfonylurea. Patients were enrolled between October 3, 2014 and December 31, 2017 (UMIN registration: #000,016,754). The #1062 study was an open-label allocation, which resulted in a marked imbalance in the numbers of enrolled patients between the two groups, making it difficult to compare the two groups. Therefore, we conducted a retrospective observational study (#2470 study) that included only those who opted for the SGLT2 inhibitor in the #1062 study.

### Inclusion and exclusion criteria

Participants in the #1062 study met the following criteria: (1) Japanese adult with type 2 (T2) DM, and (2) HbA1c (NGSP) ≥ 6.0% with diet and exercise therapy for ≥ 1 month, or with oral hypoglycemic agents other than an SGLT2 inhibitor for ≥ 1 month. Patients who showed diabetic ketoacidosis, diabetic coma, severe infection, severe trauma or malignant diseases were excluded. Women who were pregnant, possibly pregnant, or currently breastfeeding were also excluded. Patients with severe renal dysfunction, an estimated GFR (eGFR) < 30 mL/min/1.73 m^2^, or undergoing dialysis therapy were excluded. Twenty-four patients were enrolled in this study, but two patients were additionally excluded due to malignancy onset (uterine sarcoma) and the discontinuation of ambulatory visits during the follow-up period. Because only one patient was allocated to sulfonylurea in the #1062 study, the remaining 21 patients were carried over to the retrospective analysis (#2470 study).

### Study protocol

Blood and urine tests and blood pressure measurements were performed and recorded just before the start of the ipragliflozin treatment (0 M, baseline) and at 1 month (1 M) and 12 months (12 M) later. In the #1062 study, all enrolled patients were administered 50 mg of the SGLT2 inhibitor ipragliflozin daily. If the attending physician judged that HbA1c decreased poorly after 1 month, ipragliflozin could be increased to 100 mg, as it was in 4 patients. The resulting mean dose in all patients at 12 M was 52.3 mg. The eGFR was calculated by the Japanese Society of Nephrology estimating Eq. [18]. The parameters used to evaluate the effects of the SGLT2 inhibitor were ACR as a glomerular injury marker, tubular function markers including fractional excretion of sodium (FENa) and uric acid (FEUA), tubulo-interstitial injury markers including urine L-FABP and NAG to Cr ratios, urine MCP-1 as the inflammation and fibrosis marker, 8-iso prostaglandin F2α (iPF2α-III) as the oxidative stress marker, and the hemodynamics/extracellular fluid (ECF) volume marker N-terminal pro-B type natriuretic peptide (NT-proBNP). Measurements of these biomarkers were commissioned to SRL, Inc. (Tokyo, Japan).

### Statistical analyses

All statistical analyses were performed with SPSS (ver. 25.0) for Mac OS (SPSS, Chicago, IL). Data with normal distributions confirmed by the Shapiro–Wilk test are shown as means ± SDs; all other data without normal distribution are presented as medians and 1st, 3rd quantiles. For comparisons between the two paired groups, data with confirmed normal distribution were tested using a paired *t*-test, and data without confirmed normal distribution were tested using a Wilcoxon signed-rank test. For comparisons between the two unpaired groups, data with confirmed normal distribution were tested using Student's *t*-test, and data without confirmed normal distribution were tested using the Mann–Whitney *U*-test. Probability (*p*)-values < 0.05 were considered significant.

## Results

The clinical profile of the study population is provided in Table 1. The study population included many cases presenting with obesity and hypertension. Oral hypoglycemic agents were being taken by 28.6% of patients while the remaining 71.4% of patients were on diet and exercise therapy only. None of the patients was receiving insulin.

**Table 1:** Baseline characteristics of patients before starting medication with a SGLT2 inhibitor

*n*	21
Year of age	57.7 ± 14.5
Male (*n*)	12 (57.1%)
Months in clinical course	34.6 ± 28.0
Systolic blood pressure (mmHg)	131.7 ± 14.6
Diastolic blood pressure (mmHg)	81.9 ± 10.0
Body mass index (BMI, kg/m^2^)	28.3 (26.1, 31.6)
Administration of anti-hypertensive agents (%)	18 (85.7%)
Angiotensin II receptor blockers (ARB)	15 (71.4%)
Angiotensin-converting enzyme Inhibitors (ACEi)	1 (4.8%)
Calcium channel blockers (CCB)	13 (61.9%)
β-blockers	4 (19.0%)
Diuretics	5 (23.8%)
Loop diuretic	2 (9.5%)
Thiazide diuretic	3 (14.3%)
Administration of oral hypoglycemic agents	6 (28.6%)
Sulfonyl-ureas (SU)	2 (9.5%)
Dipeptidyl peptidase-4 inhibitors (DPP4i)	1 (4.8%)
Biguanides	3 (14.3%)
Thiazolidine derivatives	1 (4.8%)
α-Glucosidase inhibitors (α-GI)	1 (4.8%)
Administration of lipid-lowering agents	14 (66.7%)
Statins	14 (66.7%)

Each value represents the mean ± SD, or median, 1st quartile and 3rd quartile value

Table 2 lists the changes in parameters of the patients during the observation period. The HbA1c level was significantly decreased at 1 M and persisted until 12 M; however, HOMA-R was not changed. Although increased FEUA and decreased serum uric acid levels have been observed, this change is considered to be a common change when SGLT2 inhibitors are administered [19]. The median value of baseline eGFR was 62.6 mL/min/1.73 m^2^, and only one remarkably obese (BMI: 41.9) patient showed apparent hyperfiltration, with an eGFR ≥ 90 mL/min/1.73 m^2^. The values of eGFR in all cases were not significantly changed during the observational period. The median value of ACR was significantly decreased at 1 M; however, the significance disappeared at 12 M due to large deviations. The hemoglobin concentration was significantly increased at 1 M and 12 M.

**Table 2:** Changes in parameters in response to SGLT2 inhibitor administration

	0 M	*n*	Ipragliflozin 50 mg/day					
			1 M		*n*	12 M		*n*
BMI (kg/m^2^)	28.3 (26.1, 31.6)	21	28.0 (26.0, 32.3)		21	27.5 (25.0, 29.9)	**	21
Systolic BP (mmHg)	131.7 ± 14.6	21	127.6 ± 12.0		21	125.4 ± 15.0		21
Diastolic BP (mmHg)	81.9 ± 10.0	21	78.7 ± 8.2		21	76.2 ± 9.4	*	21
HbA1c (%)	6.60 (6.50, 7.10)	21	6.50 (6.40, 6.90)	*	21	6.50 (6.30, 6.80)	*	21
HOMA-R	3.1 (2.6, 5.6)	18	3.7 (2.8, 5.1)		20	3.9 (2.4, 9.8)		18
Hb (g/dL)	14.4 (13.3, 15.3)	19	14.6 (13.7, 15.5)	*	20	14.7 (13.9, 15.7)	*	21
ACR (mg/g Cr)	174.1 (24.3, 794.8)	21	136.0 (15.6, 268.0)	**	21	121.1 (24.5, 288.0)		21
eGFR (ml/min/1.73m^2^)	62.6 (48.4, 74.4)	21	60.4 (48.0, 74.9)		21	61.1 (45.7, 75.8)		21
NTpro-BNP (pg/mL)	39.0 (15.0, 91.0)	17	53.0 (22.5, 72.5)		20	29.0 (16.5, 47.5)		19
FENa (%)	0.89 ± 0.58	20	0.94 ± 0.61		21	1.03 ± 0.74		21
Uric acid (mg/dL)	6.40 ± 1.08	21	5.48 ± 1.35	**	21	5.57 ± 0.96	**	21
FEUA (%)	5.21 ± 2.49	20	7.03 ± 2.63	**	21	7.12 ± 2.11	**	21
Urine L-FABP/Cr (μg/g Cr)	3.83 (2.66, 6.23)	20	2.40 (1.79, 4.77)		12	2.86 (1.88, 4.08)		21
Urine NAG/Cr (U/g Cr)	7.87 (6.22, 9.96)	19	8.95 (6.30, 12.60)		20	9.04 (5.80, 12.61)		20
Urine MCP-1/Cr (pg/g Cr)	1.83 (1.10, 2.41)	19	2.34 (1.54, 3.06)		21	2.13 (1.76, 3.32)		20
Urine iPF2α-III/Cr (pg/mg Cr)	236.0 (194.5, 285.0)	20	258.0 (170.0, 358.0)		21	255.5 (212.0, 388.0)		20

Each value represents the mean ± SD, or median, 1st quartile and 3rd quartile value

*BMI* body mass index, *HOMA-R* homeostasis model assessment insulin resistance, *ACR* urine albumin-to-Cr ratio, *NTproBNP* N-terminal pro-brain natriuretic peptide, *FENa* fractional excretion of Na, *L-FABP* liver-type fatty acid binding protein, *NAG* N-acetyl-β-D-glucosaminidase, *MCP-1* monocyte chemotactic protein-1, *iPF2α-III* 8-iso prostaglandin F2α

^*^p < 0.05 vs baseline, **p < 0.01 vs baseline

We performed an analysis stratified by baseline eGFR to compare the ACR reduction between the patients with and without renal dysfunction (Table 3). In the non-renal-dysfunction (NRD) group, the median ACR value was significantly decreased by 72.7% at 1 M without eGFR changes and remained low at 12 M but not significantly. In the RD group, the mean ACR was decreased by 41.8% at 1 M, also without eGFR changes. In the NRD group, blood pressures were significantly decreased at 1 M; in the RD group they did not change significantly during the observational period. These results demonstrated that the SGLT2 inhibitor treatment resulted in an ACR reduction regardless of the presence or absence of renal dysfunction, and did not entail eGFR changes. The hemoglobin concentration was significantly increased in the NRD group but not the RD group.

**Table 3:** Reduction of ACR by SGLT2 inhibitor in two groups with higher or lower values of baseline eGFR

	0 M	*n*	Ipragliflozin					
			1 M		*n*	12 M		*n*
NRD (eGFR ≧ 60, *n* = 12)								
ACR (mg/g Cr)	163.1 (21.7, 945.8)	12	118.5 (13.0, 366.5)	*	12	114.1 (19.1, 539.4)		12
eGFR (ml/min/1.73 m^2^)	72.7 (66.4, 80.9)	12	71.0 (64.1, 80.3)		12	72.7 (62.7, 83.6)		12
Systolic BP (mmHg)	133.4 ± 13.1	12	127.0 ± 13.7	*	12	123.2 ± 12.5	*	12
Diastolic BP (mmHg)	83.8 ± 7.2	12	77.8 ± 5.7	*	12	76.8 ± 10.2		12
BMI (kg/m^2^)	28.6 (26.5, 31.1)	12	28.5 (26.2, 31.5)		12	28.5 (25.5, 29.7)	*	12
HbA1c (%)	6.9 ± 0.5	12	6.7 ± 0.5	*	12	6.6 ± 0.6	*	12
HOMA-R	4.5 (2.6, 5.6)	9	3.8 (3.1, 5.1)		12	3.6 (1.9, 4.2)		11
Hb (g/dL)	14.6 ± 1.8	11	15.1 ± 1.7	*	12	15.2 ± 1.4	*	12
RD (eGFR < 60, *n* = 9)								
ACR (mg/g Cr)	325.2 (111.0, 751.3)	9	136.0 (62.2, 264.0)	*	9	121.1 (95.9, 233.5)		9
eGFR (ml/min/1.73 m^2^)	44.4 ± 8.2	9	42.3 ± 9.8		9	44.1 ± 9.2		9
Systolic BP (mmHg)	129.3 ± 16.9	9	128.4 ± 10.0		9	128.3 ± 18.1		9
Diastolic BP (mmHg)	79.3 ± 12.9	9	79.8 ± 10.9		9	75.4 ± 8.9		9
BMI (kg/m^2^)	29.1 ± 8.1	9	28.9 ± 7.5		9	28.6 ± 7.5		9
HbA1c (%)	6.7 ± 0.4	9	6.5 ± 0.2		9	6.6 ± 0.2		9
HOMA-R	2.8 (2.8, 4.8)	9	3.5 (2.3, 10.8)		8	13.5 (6.5, 24.1)		7
Hb (g/dL)	13.6 ± 2.3	8	13.8 ± 2.1		8	14.0 ± 2.4		9

Each value represents the mean ± SD, or median, 1st quartile and 3rd quartile value

*NRD* non-renal dysfunction group, *RD* renal dysfunction group, *ACR* urine albumin-to-Cr ratio, *BMI* body mass index, *HOMA-R* homeostasis model assessment insulin resistance

^*^p < 0.05 vs baseline

To investigate the factors related to the ACR reduction, we stratified the patients into high and low ACR reduction rate groups (high and low-responders) and examined the changing rate of each parameter at 1 M according to NRD and RD designation. The rates of change of all parameters, including ACR, were analyzed with the following formula:

$${\text{Rate}}\,{\text{of}}\,{\text{change}}\,\left( {\% \Delta } \right) = 100 \times \left[ {\left( {\text{post - value}} \right) - \left( {\text{pre - value}} \right)} \right]/\left( {\text{pre - value}} \right).$$

A negative or positive value indicates a decrease or increase in the value compared to the baseline value, respectively. As shown in Table 4, a significant difference between high and low-responders was only observed in the %∆MCP-1 in the RD group.

**Table 4:** Comparison of parameter-response to SGLT2 inhibitor in groups showing high or low response of ACR at 1 M

	NRD (baseline eGFR ≥ 60)					RD (baseline eGFR < 60)				
	High responders		Low responders			High responders		Low responders		
	*n* = 6	*n*	*n* = 6		*n*	*n* = 5	*n*	*n* = 4		*n*
%∆ACR	− 62.5 ± 16.3	6	2.0 ± 35.3	**	6	− 51.5 (− 51.7, − 45.9)	5	− 27.9 (− 33.6, − 10.0)	^†^	4
%∆BMI	0.0 ± 1.7	6	− 0.8 ± 1.3		6	0.0 (− 0.6, 0.0)	5	0.0 (− 2.6, 1.2)		4
%∆systolic BP	− 7.3 ± 2.0	6	− 2.2 ± 5.0		6	− 6.5 ± 12.2	5	10.0 ± 16.1		4
%∆diastolic BP	− 8.0 ± 7.4	6	− 5.7 ± 6.9		6	− 2.5 ± 7.9	5	7.3 ± 23.8		4
%∆HbA1c	− 2.2 (− 7.1, − 1.5)	6	− 3.5 (− 4.0, 2.6)		6	− 3.9 ± 4.4	5	0.9 ± 4.6		4
%∆HOMA-R	− 36.0 ± 20.0	5	72.0 ± 131.1		4	24.1 ± 51.7	4	− 4.1 ± 22.9		4
%∆Hb	4.6 ± 4.4	5	2.8 ± 3.4		6	2.1 (0.0, 5.4)	5	0.7 (− 0.3, 0.7)		3
%∆eGFR	− 5.3 ± 10.4	6	− 0.8 ± 7.9		6	− 4.9 ± 7.5	5	− 5.4 ± 8.8		4
%∆NTproBNP	50.3 ± 56.8	5	42.7 ± 106.1		5	− 17.2 ± 31.6	4	21.3 ± 21.3		3
%∆FENa	− 29.8 (− 48.5, 36.1)	6	− 14.7 (− 34.2, 9.9)		5	7.8 ± 54.7	5	116.3 ± 176.5		4
%∆NAG/Cr	41.9 ± 72.7	6	28.5 ± 51.3		5	− 12.3 ± 26.4	5	42.7 ± 59.2		3
%∆MCP-1/Cr	16.0 ± 35.2	5	45.8 ± 83.1		5	− 25.7 ± 11.4	5	59.2 ± 17.0	^††^	4
%∆iPF2α-III/Cr	14.1 ± 36.6	5	11.2 ± 66.2		6	20.1 ± 50.4	5	− 11.4 ± 17.9		4

Each value represents the mean ± SD, or median, 1st quartile and 3rd quartile value. "%∆" indicates percent increase or decrease rate comparing to the baseline as described in the text

*NRD* non-renal dysfunction group, *RD* renal dysfunction group, *ACR* urine albumin-to-Cr rate, *BMI* body mass index, *HOMA-R* homeostasis model assessment insulin resistance, *NTproBNP* N-terminal pro-brain natriuretic peptide, *FENa* fractional excretion of Na, *NAG* N-acetyl-β-D-glucosaminidase, *MCP-1* monocyte chemotactic protein-1, *iPF2α-III* 8-iso prostaglandin F2α

^**^p < 0.01 vs high-responders in NRD, ^†^*p* < 0.05, ^††^*p* < 0.01 vs high-responders in RD

**Fig. 1:**
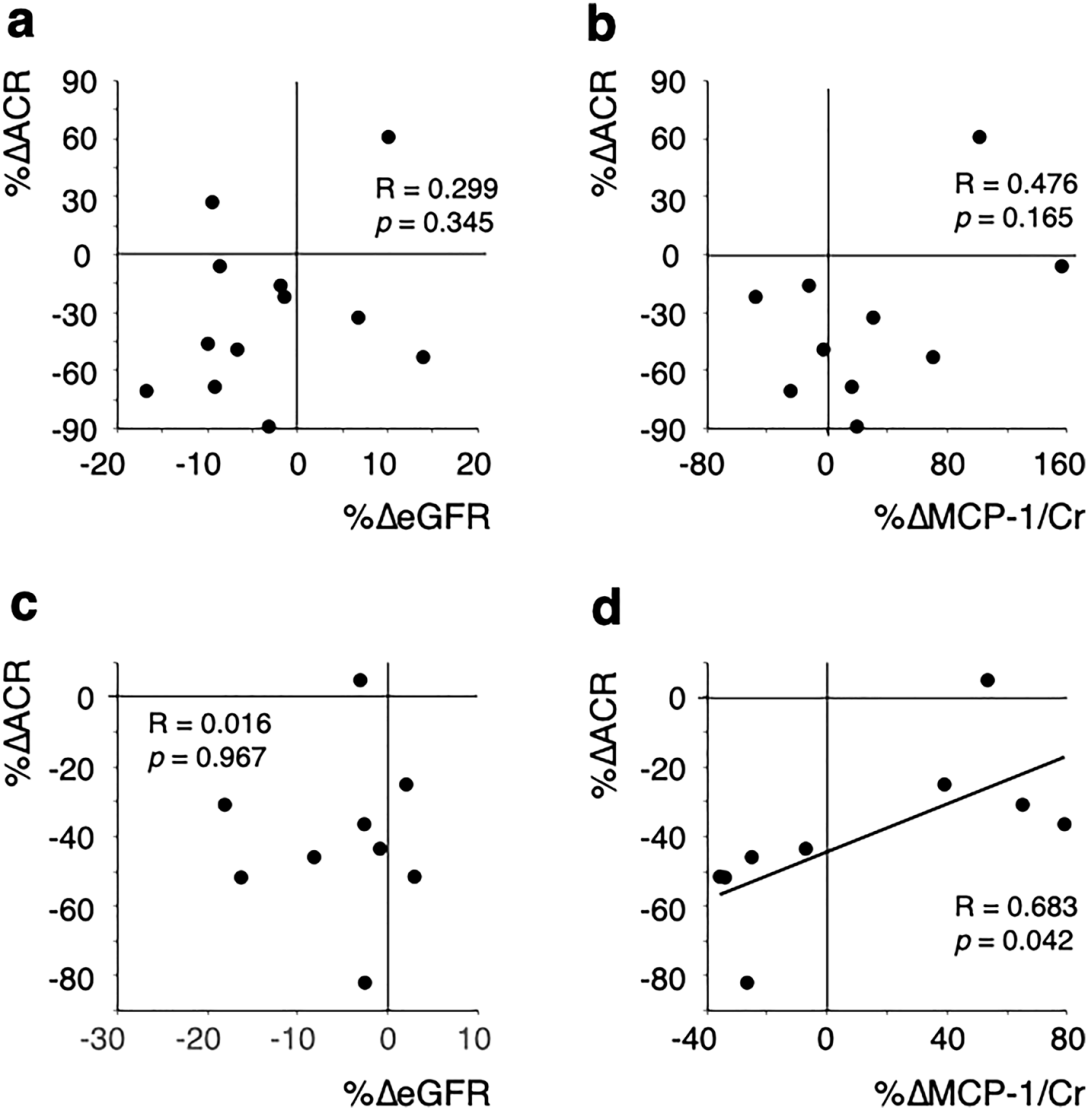
Scatter plots of selected results of the correlation analysis. Correlations of the change rate of ACR with the change rate of eGFR (**a**) and urine MCP-1 (**b**) in the non-renal-dysfunction group are depicted in the upper panel. Correlations with the change rate of eGFR (**c**) and urine MCP-1 (**d**) in the renal-dysfunction group are depicted in the lower panel. Solid lines represent linear approximation lines with statistical significance

To elucidate the relationship between the reduction of ACR and each parameter, we investigated the correlation between the ACR reduction rate and each parameter's change rate at 1 M (Table 5); the results are also depicted by scatter plot (Fig. 1). The ACR reduction rate (%∆ACR) was significantly correlated with the MCP-1 reduction rate (%∆MCP-1) in the RD group but not in NRD group, possibly indicating that among patients with renal dysfunction, more pronounced ACR reduction could be expected in those with greater reduction in MCP-1.

**Table 5:** Correlation analysis of the changes in the multiple parameters with the reduction of ACR at 1 M

	NRD (eGFR ≥ 60, *n* = 12)			RD (eGFR < 60, *n* = 9)		
vs %∆ACR	*R*	*p*-value	*n*	*R*	*p*-value	*n*
%∆BMI	− 0.100	*n.s*	12	0.236	*n.s*	9
%∆systolic BP	0.354	*n.s*	12	0.578	*n.s*	9
%∆diastolic BP	− 0.005	*n.s*	12	0.578	*n.s*	9
%∆HbA1c	0.475	*n.s*	12	0.386	*n.s*	9
%∆HOMA-R	0.464	*n.s*	9	− 0.320	*n.s*	8
%∆Hb	− 0.103	*n.s*	11	0.087	*n.s*	8
%∆eGFR	0.299	*n.s*	12	0.016	*n.s*	9
%∆NTproBNP	0.474	*n.s*	10	0.723	*n.s*	7
%∆FENa	0.194	*n.s*	11	0.200	*n.s*	9
%∆NAG/Cr	0.137	*n.s*	11	0.179	*n.s*	8
%∆MCP-1/Cr	0.476	*n.s*	10	0.683	0.042	9
%∆iPF2α-III/Cr	− 0.006	*n.s*	11	− 0.156	*n.s*	9

*NRD* non-renal dysfunction group, *RD*: renal dysfunction group, *R* Pearson’s correlation coefficient, *BMI* body mass index, *HOMA-R* homeostasis model assessment insulin resistance, *ACR* urine albumin-to-Cr ratio, *NTproBNP* N-terminal pro-brain natriuretic peptide, *FENa* fractional excretion of Na, *NAG* N-acetyl-β-D-glucosaminidase, *MCP-1* monocyte chemotactic protein-1, *iPF2α-III* 8-iso prostaglandin F2α, *n.s.* not significant

## Discussion

In the present study, we confirmed that the ACR was significantly reduced after administration of an SGLT2 inhibitor in diabetic patients with or without renal dysfunction. The ACR reduction was correlated with the reduction of urine MCP-1 in the RD group, independently of other factors including obesity, systemic blood pressure and hyperglycemia, whereas no parameter showed a significant correlation in the NRD group. These results suggest that the underlying mechanism of ACR reduction by SGLT2 inhibitor in RD patients might be different from that of patients without renal dysfunction, and its effect on tubulo-interstitial disorders might play a key role in the ACR reduction of patients with RD.

It is generally believed that TG feedback resetting is believed to be one of the principal mechanisms underlying the amelioration of albuminuria and renal damage progression by SGLT2 inhibitors, however there was no difference in eGFR related with ACR reduction in our study. However, few cases presented with clear GHF in our population, and the median eGFR of NRD group was 72.7 mL/min/1.73 m^2^. This may be the principal reason why no significant change in eGFR was observed even in NRD group in the present study, and it does not seem to deny the effectiveness of SGLT2 inhibitor in improving GHF.

Tubulo-interstitial disorders are closely involved in the progression of chronic kidney disease. Persistent albuminuria induces inflammation and chemokine secretions in the proximal tubules, and it finally accelerates fibrosis. In diabetes, excess filtered glucose is reabsorbed primarily through SGLT2, resulting in its accumulation in the tubular cells and surrounding interstitium. Accordingly, the accumulated glucose accelerates the production of AGEs, leading to chronic inflammation and oxidative stress and finally to fibrosis [5, 20, 21]. These tubulo-interstitial disorders related to high glucose loading in diabetic kidneys are known as diabetic tubulopathy, which seems to be one of the central pathogeneses of renal dysfunction in diabetes.

It has been expected that SGLT2 inhibitors inhibit tubular glucose accumulation and the progression of tubulopathy. Indeed, SGLT2 inhibitors have been shown to ameliorate renal interstitial fibrosis more effectively than other oral hypoglycemic agents or insulins [22, 23]. The SGLT2 inhibitor empagliflozin was reported to inhibit the inflammatory cascade by restoring the impaired mitochondria function and autophagy in human tubular cells cultured in high-glucose conditions [24]. The anti-fibrotic effect of SGLT2 inhibitors is not limited to patients with diabetes; an animal study using unilateral ureteral obstruction, a typical renal fibrosis model, showed that use of an SGLT2 inhibitor inhibits fibrosis via a variety of fibrotic pathway-related mechanisms involving TGF-β1, αSMA, the Wnt cascade, CTGF, and fibronectin [25]. The anti-fibrotic effect of an SGLT2 inhibitor on tubulo-interstitial disorders is pleiotropic, and inhibitions of the AGEs accumulation and oxidative stress due to reduced ATP consumption have also been demonstrated to be involved [15, 26].

In this study, we observed correlations in the RD group between ACR and MCP-1 reductions of urinary excretion, but not between ACR and GFR, ECF volume, or salt excretion. MCP-1 is a molecule that drives functional transformation in leukocytes responding to inflammation [27]. Our present results might indicate that the amelioration of interstitial inflammation by the administration of SGLT2 inhibitor is involved in ACR reduction in those patients. Terami et al. demonstrated that the SGLT2 inhibitor dapagliflozin inhibited interstitial fibrosis in a dose-dependent manner in diabetic mice, and in cultured cells dapagliflozin dose-dependently inhibited macrophage infiltration and the expressions of TGF-ß, intercellular adhesion molecule-1 (ICAM-1) and MCP-1, which are up-regulated in high-glucose conditions, and concomitantly inhibited the gene expression of inflammatory cytokines and oxidative stress [28]. Ishibashi et al. also show in cultured cells that MCP-1 expression induced by high-glucose condition is suppressed by tofogliflozin in a dose-dependent manner, and at the same time, ROS production and apoptosis are also suppressed [29].

These results indicate that the inhibition of urinary MCP-1 excretion would indeed reflect the amelioration of tubulo-interstitial disorders by SGLT2 inhibitors. This study’s finding of a correlation of ACR reduction with MCP-1 reduction, but not with eGFR or ECF volume, might indicate that ACR reduction by SGLT2 inhibitor is predominantly derived from the amelioration of tubulo-interstitial disorders in RD patients. Although there are some cases in which MCP-1 excretion is increasing while ACR is decreasing in the population, changes may be based on other factors, such as correcting glomerular hypertension and reducing extracellular fluid volume. However, even in such cases, the inhibition of interstitial damage or its reduced progression seems to have a certain effect on the reduction of ACR, which may contribute to the significant association between the reduction of MCP-1 excretion and the reduction of ACR in the entire RD group. The mechanism by which improved tubulo-interstitial disorders resulted in the reduction of ACR might be a consequence of improved tubular albumin-handling capability. In addition, it has also been reported that tubular cell damage causes changes in the slit membrane function of glomerular podocytes and exacerbates albuminuria through changes in the tubular expression of sirtuin 1 and nicotinamide mononucleotide. The results referred above have indicated that tubulo-interstitial disorders may cause the podocyte damage and resultant albuminuria [30, 31]. Thus, we speculate that reduced proximal tubule-to-podocyte cross-talk associated with reduced interstitial damage, as well as restored proximal tubular albumin-handling capacity, may contribute to the association between reduced MCP-1 excretion and reduced ACR. To validate this speculation, further basic and clinical studies are required, including an investigation of the changes in the tubulo-interstitial parameters after SGLT2 inhibitor administration in non-diabetic patients with proteinuria.

Our study has several limitations. Since this was a prospective observational study and no randomized controlled trial was conducted, conclusions regarding the efficacy of the SGLT2 inhibitor cannot be made. This study focused on differences in the underlying mechanisms between RD and NRD patients. In addition, this was a single-center small-scale study, and the small patient population may have influenced the results. However, renal dysfunction and non-dysfunction patients each accounted for roughly half of the patients, which may be a reasonable population for comparisons between these two groups.

## Conclusions

**Fig. 2:**
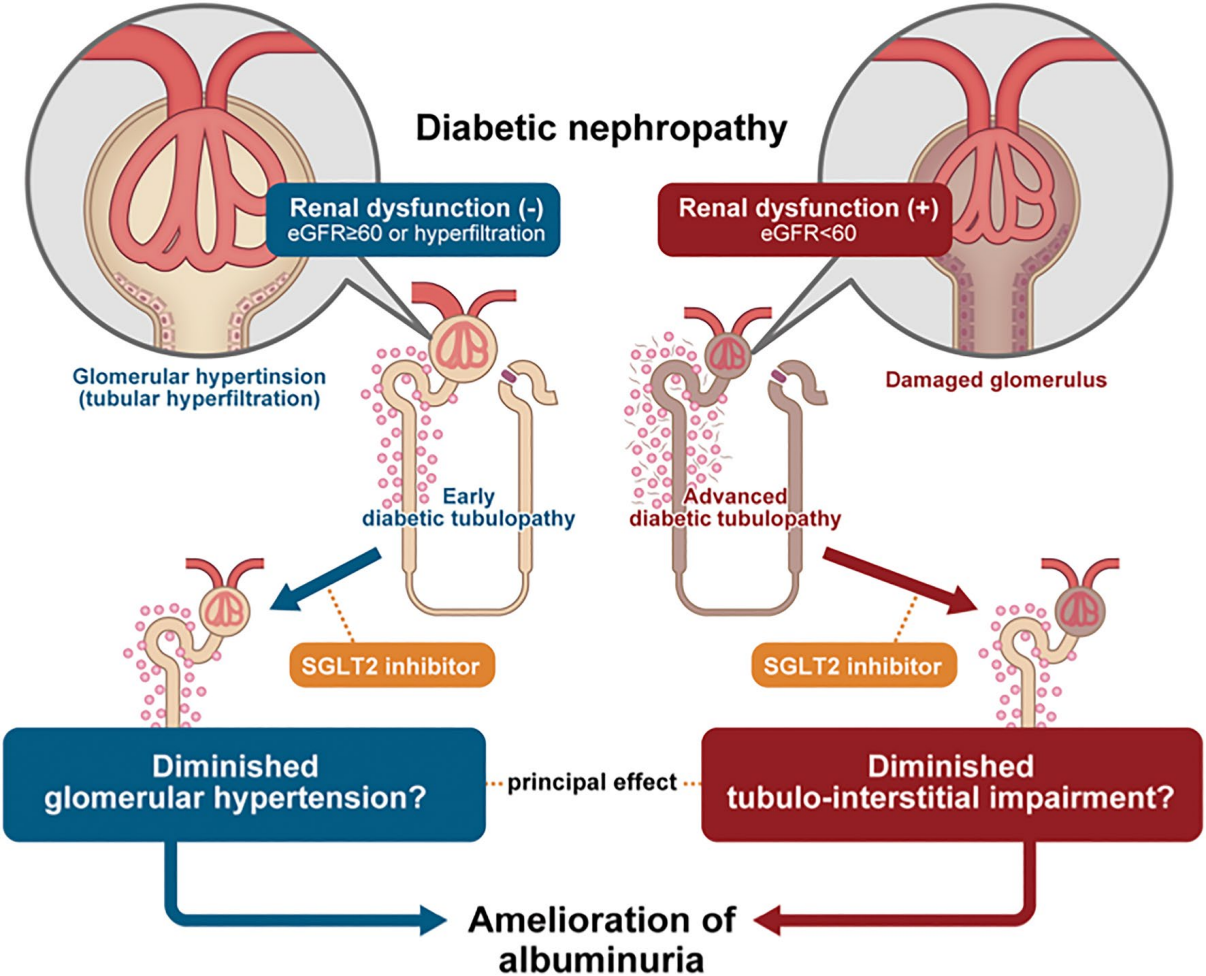
Theoretical schema of the underlying mechanism of SGLT2 inhibitors in patients with and without renal dysfunction. In early diabetes, patients do not show renal dysfunction, and glomerular hypertension due to hyperfiltration is generally observed. In this period, interstitial injury around the proximal tubule is typically only mild, and peritubular glucose and AGEs accumulation (represented by small red circles) is often limited (left panel). In more advanced periods of diabetes, glomerular damage becomes more pronounced and GFR also decreases. Accumulation of glucose and AGEs to the tubulo-interstitial tissue advances, and tubulo-interstitial damage and fibrosis progress (right panel)

In conclusion, the SGLT2 inhibitor ipragliflozin showed anti-albuminuric effects regardless of the presence/absence of renal dysfunction; however, this effect may differ according to the presence or absence of renal dysfunction. The hypothesis we consider from our results is shown in Fig. 2. In patients with renal dysfunction, its effect on glomerular hyperfiltration might become less advantageous, and the ameliorating effect on the tubulo-interstitial disorders might be more critical to the anti-albuminuric effect of SGLT2 inhibitors.

## Data Availability

The datasets used and/or analyzed during the current study are available from the corresponding author on reasonable request.
